# Immune-Mediated Disease Flares or New-Onset Disease in 27 Subjects Following mRNA/DNA SARS-CoV-2 Vaccination

**DOI:** 10.3390/vaccines9050435

**Published:** 2021-04-29

**Authors:** Abdulla Watad, Gabriele De Marco, Hussein Mahajna, Amit Druyan, Mailam Eltity, Nizar Hijazi, Amir Haddad, Muna Elias, Devy Zisman, Mohammad E. Naffaa, Michal Brodavka, Yael Cohen, Arsalan Abu-Much, Muhanad Abu Elhija, Charlie Bridgewood, Pnina Langevitz, Joanna McLorinan, Nicola Luigi Bragazzi, Helena Marzo-Ortega, Merav Lidar, Cassandra Calabrese, Leonard Calabrese, Edward Vital, Yehuda Shoenfeld, Howard Amital, Dennis McGonagle

**Affiliations:** 1 Department of Medicine ‘B, Zabludowicz Center for Autoimmune Diseases, Sheba Medical Center, Tel-Hashomer 10457, Israel; watad.abdulla@gmail.com (A.W.); yehuda.shoenfeld@sheba.health.gov.il (Y.S.); Howard.Amital@sheba.health.gov.il (H.A.); 2 Sackler Faculty of Medicine, Tel-Aviv University, Tel-Aviv 69978, Israel; hmahajna@gmail.com (H.M.); Amit.Druyan@sheba.health.gov.il (A.D.); michal.brodavka@sheba.health.gov.il (M.B.); Yael.cohen2@sheba.health.gov.il (Y.C.); Pnina.Langevitz@sheba.health.gov.il (P.L.); Merav.Lidar@sheba.health.gov.il (M.L.); 3 Rheumatology Unit, Sheba Medical Center, Tel-Hashomer 10457, Israel; 4 NIHR, Leeds Biomedical Research Centre, The Leeds Teaching Hospitals NHS Trust & Leeds Institute of Rheumatic and Musculoskeletal Medicine, University of Leeds, Leeds LS9 7TF, UK; G.DeMarco@leeds.ac.uk (G.D.M.); C.D.Bridgewood@leeds.ac.uk (C.B.); h.marzo-ortega@leeds.ac.uk (H.M.-O.); E.M.J.Vital@leeds.ac.uk (E.V.); 5 Gastroenterology Department, Sheba Medical Center, Tel-Aviv 10457, Israel; 6 Department of Neurology, Sheba Medical Center, Tel-Aviv 10457, Israel; mailameltity@gmail.com; 7 Rheumatology Unit, Carmel Medical Center, Michal Street, Haifa 3436212, Israel; NizarHi@clalit.org.il (N.H.); haddadamir@yahoo.com (A.H.); nelias1@bezeqint.net (M.E.); devyzisman@gmail.com (D.Z.); Mhndhija100@gmail.com (M.A.E.); 8 Ruth and Bruce Rappaport Faculty of Medicine, Technion-Israel Institute of Technology, Haifa 3200003, Israel; 9 Department of Rheumatology, Galilee Medical Center, Azrieli Faculty of Medicine, Bar-Ilan University, Safed 22100, Israel; MohammadN@gmc.gov.il; 10 Leviev Heart Center, Sheba Medical Center, Tel Hashomer, Tel Aviv 10457, Israel; arsalanabumuch1@gmail.com; 11 Department of Rheumatology, Mid Yorkshire Hospitals, West Yorkshire WF8 1PL, UK; joanna.mclorinan1@nhs.net; 12 Centre for Disease Modelling, Department of Mathematics and Statistics, York University, Toronto, ON M3J 1P3, Canada; 13 Fields-CQAM Laboratory of Mathematics for Public Health (MfPH), York University, Toronto, ON M3J 1P3, Canada; 14 Cleveland Clinic Foundation, 9500 Euclid Avenue, Desk A50, Cleveland, OH 44195, USA; calabrc@ccf.org (C.C.); calabrl@ccf.org (L.C.)

**Keywords:** vaccine safety, COVID-19, mRNA-based vaccine, adenoviral vector-based vaccine, immune-mediated diseases

## Abstract

Background: Infectious diseases and vaccines can occasionally cause new-onset or flare of immune-mediated diseases (IMDs). The adjuvanticity of the available SARS-CoV-2 vaccines is based on either TLR-7/8 or TLR-9 agonism, which is distinct from previous vaccines and is a common pathogenic mechanism in IMDs. Methods: We evaluated IMD flares or new disease onset within 28-days of SARS-CoV-2 vaccination at five large tertiary centres in countries with early vaccination adoption, three in Israel, one in UK, and one in USA. We assessed the pattern of disease expression in terms of autoimmune, autoinflammatory, or mixed disease phenotype and organ system affected. We also evaluated outcomes. Findings: 27 cases included 17 flares and 10 new onset IMDs. 23/27 received the BNT - 162b2 vaccine, 2/27 the mRNA-1273 and 2/27 the ChAdOx1 vaccines. The mean age was 54.4 ± 19.2 years and 55% of cases were female. Among the 27 cases, 21 (78%) had at least one underlying autoimmune/rheumatic disease prior the vaccination. Among those patients with a flare or activation, four episodes occurred after receiving the second-dose and in one patient they occurred both after the first and the second-dose. In those patients with a new onset disease, two occurred after the second-dose and in one patient occurred both after the first (new onset) and second-dose (flare). For either dose, IMDs occurred on average 4 days later. Of the cases, 20/27 (75%) were mild to moderate in severity. Over 80% of cases had excellent resolution of inflammatory features, mostly with the use of corticosteroid therapy. Other immune-mediated conditions included idiopathic pericarditis (*n* = 2), neurosarcoidosis with small fiber neuropathy (*n* = 1), demyelination (*n* = 1), and myasthenia gravis (*n* = 2). In 22 cases (81.5%), the insurgence of Adverse event following immunization (AEFI)/IMD could not be explained based on the drug received by the patient. In 23 cases (85.2%), AEFI development could not be explained based on the underlying disease/co-morbidities. Only in one case (3.7%), the timing window of the insurgence of the side effect was considered not compatible with the time from vaccine to flare. Interpretation: Despite the high population exposure in the regions served by these centers, IMDs flares or onset temporally-associated with SARS-CoV-2 vaccination appear rare. Most are moderate in severity and responsive to therapy although some severe flares occurred. Funding: none.

## 1. Introduction

Since its initial outbreak in late December 2019, the “Severe Acute Respiratory Syndrome-related Coronavirus type 2” (SARS-CoV-2) infection has resulted in over 3.1 million deaths and has contributed to immeasurable additional medical and economic consequences due to the lockdown measures designed to control the virus spread and reduce disease mortality [1]. The strategy to end the “Coronavirus Disease 2019” (COVID-19) pandemic rests almost entirely on the few drugs available approved by regulatory bodies, such as the United States of America (US) Food and Drug Administration (FDA), like Remdesivir [2], and on vaccines, which, as a result, have been developed rapidly through global collaborations and unprecedented efforts [3,4].

While vaccinations usually represent a safe and effective tool against infectious diseases, several critical steps of their development present distinct challenges. The US Centers for Disease Control and Prevention (CDC) have identified six stages: namely, the (i) exploratory, (ii) pre-clinical, (iii) clinical development, (iv) regulatory review and approval, (v) manufacturing, and (vi) quality control phases [5]. Consequently, achieving a safe, effective vaccine is a time- and resource-consuming process that ordinarily takes up to 10–15 years on average to be successfully completed. Conversely, in a rapidly evolving pandemic, such a protracted process may cost many more lives than it could save. Hence, due to the emergency situation related to the COVID-19 pandemic, several steps of the path to prevention have been fast-tracked. This has increased uncertainties about the long-term efficacy and safety of vaccines [6]. This abbreviated development process, therefore, warrants an increased scrutiny in post-marketing surveillance of biologically plausible side effects. Among these are immune-mediated phenomena.

To date, authorized COVID-19 vaccine products and those pending approval have shown good efficacy, excellent safety, and tolerability in randomized clinical trials (RCTs) [7,8,9,10,11,12]. Additionally, the first SARS-CoV-2 vaccine to the market—BNT162b2 (Pfizer/BioNTech)—has seen high utilization in several jurisdictions including the United Kingdom (UK), Israel and the USA, where vaccine roll-out commenced in early-to mid-December 2020. Although millions of individuals have been vaccinated, there have been so far few reports of vaccine-associated immune-mediated disease (IMD) development, with the only notable exception of rare, potentially immune-mediated autoimmune thrombosis [13].

The role of infectious agent derived antigens, including SARS-CoV-2 [14], as an IMD trigger, is well recognized with both natural infection or, less commonly, after vaccination. An array of innate and adaptive immune mechanisms, including molecular mimicry, may be responsible for these phenomena [15,16]. Adjuvants are a group of substances that drive innate immune system pattern recognition receptor (PRR) activation. They are commonly used in vaccines to boost immune reactivity towards target antigens [17,18]. A link between disorders that are characterized by innate and adaptive immune system dysregulation and exposure to an adjuvant such as aluminum, and squalene, has been reported [19,20,21]. These disorders, together with others, encompass a broad spectrum of reactions, which collectively have been referred to as the “vaccine adjuvant-related syndrome”, term coined by Gherardi in 2003 [22] and, subsequently, as the “Autoimmune/Autoinflammatory Syndrome Induced by Adjuvants” (ASIA syndrome) [23]. However, these two syndromes remain highly controversial entities [24,25].

The adjuvanticity of the available SARS-CoV-2 vaccines is novel and, to a large degree, depends on the intrinsic adjuvanticity of vaccine messenger RNA (mRNA) or DNA which, respectively, stimulates the innate immunity through endosolic and cytoplasmic nucleic acid receptors such as Toll-Like Receptors (TLRs) 3, 7, 8, and 9, and components of the inflammasome, including Retinoic acid-Inducible Gene I (RIG-I) and Melanoma Differentiation-Associated Gene 5 (MDA5) [26,27].

Several IMDs, most notably autoimmune connective tissue diseases, may be linked to altered nucleic acid metabolism and processing, which have been shown to stimulate TLR-7 and TLR-9 experimentally and in humans [28,29]. However, vaccine development programs are not generally performed on subjects with IMDs where issues such as disease activity and immunotherapy may alter the course of disease. These diseases are too uncommon for such events to be detected in standard vaccine trial populations [30]. They affect about 3–7% of the general population with an estimated incidence of 80 per 10^5^ person-years [30]. Moreover, vaccine safety evaluation is generally considered as a secondary objective, with pivotal RCTs being underpowered to enable statistical analyses of endpoints of specific side effects, including IMDs [31]. Further, some adverse events could be hard to identify, especially those of vaccines produced utilizing new technologies [31].

During a pandemic, it is of paramount importance to provide real-time data concerning vaccine safety. Exchange of information, even though preliminary, can shed light on potential mechanisms underlying vaccine action [31] and generate hypotheses that can be further tested by ad hoc, large epidemiological surveys. Frontline physicians and public health-workers, who are the point of first contact in case of insurgence of an adverse event, can play a key role in this.

There are two major ways for identifying and reporting side effects following vaccination: namely, active, or passive surveillance. The former generally implies data mining of large electronic health record systems and is able to capture more adverse events than passive surveillance, even if most of them are usually mild. The latter system, being on a voluntary basis, is biased towards the reporting of more severe and earlier adverse events and can have multiple shortcomings, such as under-reporting of mild symptoms, unconfirmed diagnosis, and not always clear temporal links between vaccine administration and symptoms development. Even if the system generated by passive surveillance constitutes the backbone of the system exploited by active surveillance, only the latter can enable a rigorous analysis and comparison of epidemiological trends (i.e., incidence/prevalence rates) of adverse events following immunization, in terms of spatio-temporal trend and stratifying them according to the vaccination status [31].

Given the constantly evolving situation and the uncertainty about the still ongoing COVID-19 pandemic, it is crucial to collect data directly from frontline physicians [32].

Accordingly, we evaluated clinical networks in three countries at the vanguard of SARS-CoV-2 vaccination programs looking for evidence of new-onset or flare of immune disease temporally linked to vaccine administration, collecting data from collaborating physicians, utilizing reliable, confirmed diagnosis, and including also 1-month delayed reported symptoms.

## 2. Methods

This study was reported according to the “CAse REports” (CARE) guidelines [33]. Verbal or written consent was obtained from all patients for the anonymized use of data. Data were obtained from three countries, Israel (Sheba Medical Centre in Tel Aviv, Carmel Hospital in Haifa, and Galilee Medical Centre in Nahariya), UK (The Leeds Teaching Hospitals NHS Trust) and USA (Cleveland Clinic). All patient events reported occurred in the study period December 2020 to February 2021.

We identified patients presenting with IMD, especially rheumatic diseases, including new presentations, new disease relapses or severe disease worsening that developed shortly after vaccination. Given that the most commonly recognized post infectious disease and post vaccination type of arthritis, termed reactive arthritis, may be diagnosed when onset is within one month of infection exposure, we took a cut-off of 28 days post vaccination as relevant.

According to the World Health Organization (WHO) guidelines [34], “Adverse Events Following Immunization” (AEFI) can be defined as any untoward, unfavorable, or unintended medical occurrence (ranging from indication, symptom, series of symptoms, disease, to abnormal laboratory findings) following vaccination, not necessarily exhibiting a consistent, causal relationship with the administration of the vaccine product. Based on the nature of the causal association, AEFIs can be classified into: (i) vaccine product-, (ii) vaccine quality defect-related reactions, (iii) immunization error or anxiety, and (iv) co-incidental event. Vaccine can act either as a factor or co-factor together with underlying predisposing conditions related to the biological or genetic make-up of the individual and the interaction of environmental variables. AEFIs are, as such, concrete examples of complex, multi-factorial events [35].

**Figure 1:**
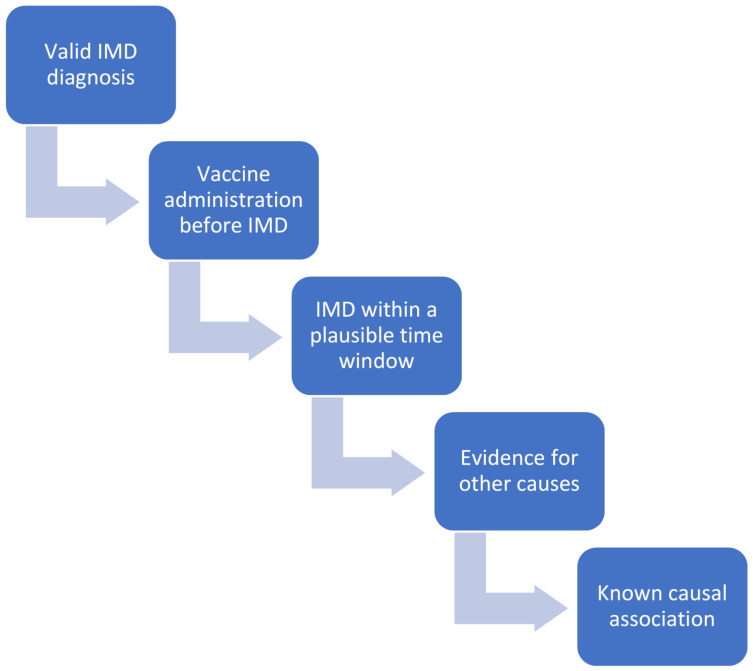
Causality assessment of “Adverse Events Following Immunization” (AEFIs) after “Coronavirus Disease 2019” (COVID-19) vaccination based on the World Health Organization (WHO) guidelines, which propose a comprehensive four-step analytical and algorithmic diagramming process (namely, evaluation and assessment of (i) the temporal association between vaccine administration and AEFI/immune-mediated disease (IMD); (ii) a plausible time window between vaccine administration and AEFI/IMD; (iii) other causes, such as underlying co-morbidities or drugs taken by the patient, which could explain the insurgence of AEFI/IMD; and, (iv) strength of the causal association, based on what is currently known from the literature).

We evaluated the causality of the AEFI after COVID-19 vaccination based on the WHO guidelines, which propose a comprehensive four-step analytical and algorithmic diagramming process. Even though the WHO instrument has been criticized [35], currently, there exist no valid and reliable alternatives [36]. All possible “other causes” that could explain the insurgence of the AEFI, excluding the etiopathological role of the vaccine, were initially considered. Notably, all patients were well controlled and reported general well-being with pharmacological therapy prior to vaccination, with the exception of a case in which the administration of rituximab was delayed for immunization purpose. After validating IMD diagnosis, and excluding non-vaccination related causalities, biological plausibility and temporal compatibility between the immunization and the occurrence of the AEFI were assessed (Figure 1). To ensure a reliable assessment of AEFIs, a multi-disciplinary evaluation was performed, involving different specialists, ranging from immunologists, rheumatologists, internal medicine doctors and epidemiologists, as recommended by the WHO guidelines. Since these guidelines and checklists concern general AEFIs and not particular classes of adverse events after immunization, similarly to the approach outlined in [36], we have adapted them to the specific case of IMD-like side effects (Figure 1).

Moreover, based on the type of IMD, AEFIs were categorized according to the immunologic disease continuum for the classification of vaccine reactions proposed by Koenig et al. [37].

This scheme includes the following categories including “classical adaptive immune-meditated diseases” (mainly involving B- and T-cells and primary lymphoid organs), “innate immune-mediated diseases” (affecting cells of the innate immune system) and “intermediate diseases” (including major histocompatibility complex (MHC) class I-related disease), “non-immunoglobulin E (IgE)- and IgE-related hypersensitivity” and “innate immune driven” or autoinflammatory diseases as earlier proposed [38].

Descriptive statistics were used to summarize the age, sex, history of IMD, the average of onset of symptoms, severity, therapeutics administered, and key clinical and laboratory findings.

## 3. Results

We identified 27 cases of IMDs (15 females, 55.6%; 12 males, 44.4%; mean age 54.44 ± 19.20 years). 21 had an autoimmune/rheumatic background. This could be further subcategorized as autoimmune (*n* = 11, 52.4%), autoinflammatory (*n* = 4, 19.0%) and mixed pattern (*n* = 6, 28.6%) disorders before COVID-19 vaccination. In six patients (22.2%), there was no autoimmune/rheumatic background and patients presented new-onset rheumatic and musculoskeletal (RMD) and non-RMD disorders (Table 1).

Other factors such as alternative infectious triggers, active COVID-19 infection around vaccination, joint trauma or major surgery as triggering events were not found, although patients were not systematically tested for SARS-CoV-2 infection unless there were typical symptoms.

Twenty-three (85.2%), two (7.4%), and two (7.4%) received the BNT-162b2, mRNA-1273 and ChAdOx1 vaccines, respectively. Twenty cases (74.1%) were reported in Israel, five (18.5%) in the UK, and two (7.4%) in the USA.

The average time between vaccination and new-onset or flare of symptoms was 4 days (median of 4 days [1–25 days] in those who developed an IMD after the first dose and a median of 4 days [1–7 days] in those after the second dose) with most cases occurring after the first inoculation (77.8%). Twelve cases flared after the first vaccine dose, one case flared after both the first and the second vaccine dose, and four cases flared only after the second vaccine dose. Fifteen cases (55.6%) did not receive the second vaccination.

Although we did not formally collect data on well-known vaccine adverse reactions such as fever, headaches, myalgia, and arm pain following vaccination, these features did not appear to be severe from review of medical records. Most IMDs were mild-to-moderate in terms of severity (*n* = 20, 74.1%).

In 22 cases (81.5%), the development of AEFI/IMD could not be explained based on the drug received by the patient. In 23 cases (85.2%), AEFI development could not be explained based on the underlying disease/co-morbidities. Only in one case (3.7%), the timing window of the insurgence of the side effect was considered not compatible with the time from vaccine to flare (Table 1).

### 3.1. RMD Cases

The individual cases are summarized in Table 2.

Overall, 20/27 cases had RMD disease. Among those, 11 patients had arthritis, 9 flares, and 2 of new-onset, and in 8/11 patients, arthritis was the only presentation without extra-articular features, 7 cases of arthritis were seronegative inflammatory arthritis and 3 were anti-citrullinated protein antibody positive (ACPA+), rheumatoid factor positive (RF+) autoimmune and 1 case of mixed pattern nature (Figure 1). One case of a flare in gout was noted with flare in this arthropathy being well reported with native SARS-CoV-2 infection during the first wave of the pandemic [39].

**Figure 2:**
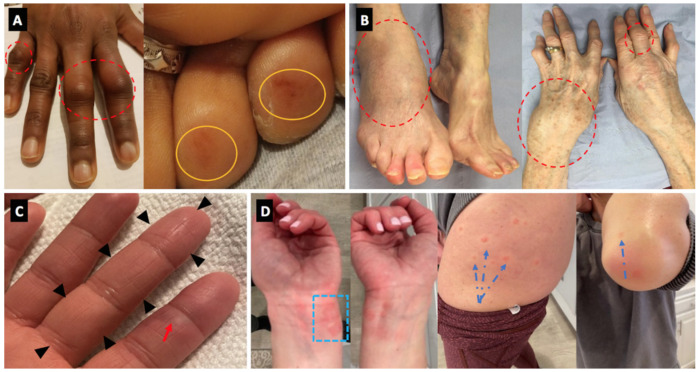
Panel (**A**) (Case 2)—Florid clinical arthritis of the proximal interphalangeal joints (dotted red circles). Concomitant, painless rash on two toe tips (orange circles). Panel (**B**) (Case 23)—Severe articular swelling of the right ankle, left wrist, and the small joints of the fingers (dotted red circles). Panel (**C**) (Case 9)—dactylitis of the third finger (black arrowheads) associated with chilblain lesion of the second finger (red arrow). Panel (**D**) (Case 10)—chilblain-like lesions of the fingers and palms, associated with urticarial lesions of the wrists (blue dotted square), thighs and elbows (blue dotted arrows).

Four cases of Behcet’s disease (BD) flare were noted in Israel with all of these having oral ulceration flares with one experiencing associated arthritis. None of the BD cases experienced major internal organ flares or immunothrombotic vascular episodes with disease limited to cutaneous and articular flares (Figure 2).

**Figure 3:**
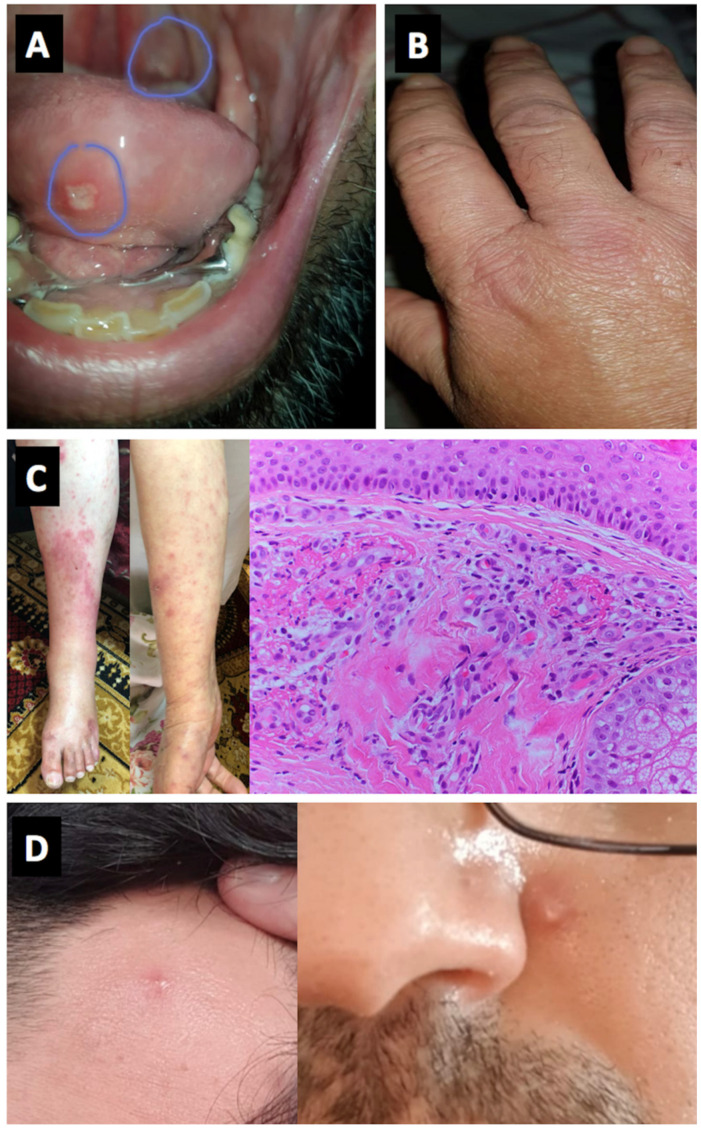
Panels (**A**,**B**) (Cases 11 and 12)—Oral aphthous lesions and small joints arthritis, Behçet’s disease. Panel (**C**) (Case 22)—Vasculitic lesions of the skin on lower limbs and forearms. Histology consistent with fibrinoid necrosis of small vessels. Panel (**D**) (Case 17)—Pustular skin lesions on the forehead and face, Behçet’s disease.

With respect to autoimmune connective tissue diseases, 3 cases of systemic lupus erythematosus (SLE) (2 flares and one case with predominant discoid SLE clinical features that converted to confirmed pleuropericarditis) were noted. A single case of known dermatomyositis experienced a flare in cutaneous disease but not myositis following vaccination (Table 2) (Figure 3B).

Three vasculitis cases, all new-onset were reported including a Henoch-Schönlein Purpura (HSP) pattern, and 2 cases of chilblain lesions (Figure 2).

One new case of polymyalgia rheumatica (PMR) was reported and 1 case of remitting seronegative symmetrical synovitis with pitting edema (RS3PE) (new-onset) was documented in a subject with confirmed PMR that was treated 15 years earlier. No cases of Giant cell arteritis (GCA) were noted.

### 3.2. Non-RMD

**Figure 4:**
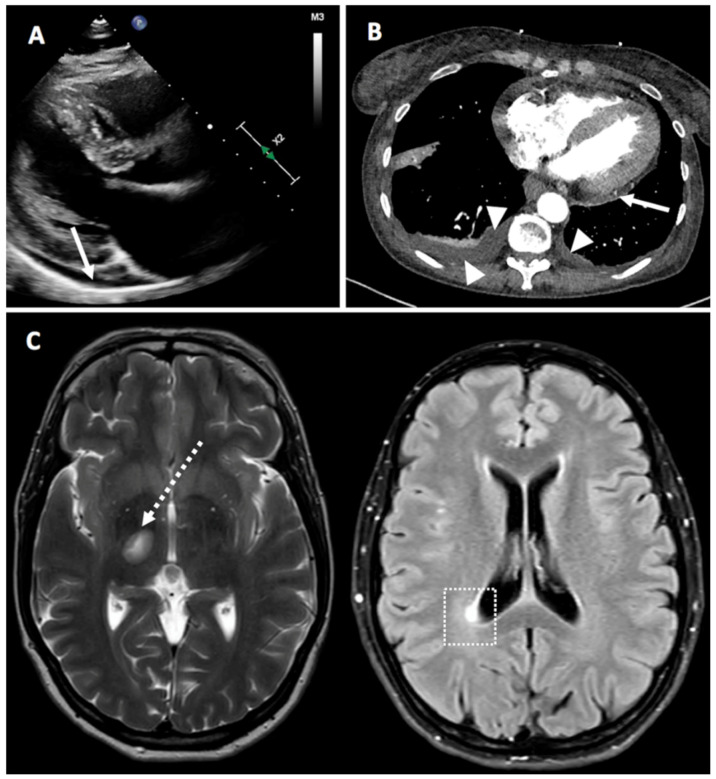
Panel (**A**) (Case 4)—Parasternal, long axis view from a 2-D echocardiogram showing a small-sized posterior pericardial effusion (white arrow). Panel (**B**) (Case 27)—Computerized tomography scan demonstrating posterior pericardial effusion (white arrow) and bilateral pleural effusion (white arrowheads). Panel (**C**) (Case 7)—Magnetic resonance imaging of the brain showing demyelinating lesions in the left mesencephalic (dotted white arrow) and in the right peri-ventricular occipital white matter (white dotted square).

The individual cases are summarized in Table 2. In our study, 7 non-RMD cases were reported. Among these, 4 autoimmune neurological disorders were found. Two new-onset myasthenia gravis cases occurred, both after the second dose of BNT162b2 vaccine, one being severe. A single new-onset case of multiple sclerosis (MS) was reported after a week after the first dose of BNT162b2 vaccine, which was clinically moderate and responded well to therapy (Figure 4, in particular Figure 4C). One moderate flare case of neurosarcoidosis and small fiber neuropathy that resolved spontaneously was also reported.

We observed two cases of pericarditis that we considered to be more autoinflammatory than autoimmune (Figure 4, in particular Figure 4A,B). Notably, one case was particularly suggestive of vaccine-induced inflammation as with each vaccination, pericarditis occurred 4 days after the first dose and a flare 4 days after the second dose. Both episodes were treated with nonsteroidal anti-inflammatory drugs (NSAIDs) and colchicine and responded well.

An additional case of pericarditis flare was noted in an ACPA+ RF+ RA case but there was no flare in joint disease. Miscellaneous diagnosis cases included a case of ulcerative colitis (UC) and severe associated hypereosinophilic syndrome that flared.

### 3.3. Laboratory Findings

Among 11 cases of arthritis, only 3 were of autoimmune (RF was positive in two cases and 1 was anti-nuclear antibody or ANA positive). ACPA was positive in only one case of pericarditis but with no particular manifestation. CRP and ESR were generally high. Other laboratory tests are reported in Table 1.

### 3.4. Therapy

As outlined in Table 1 and Table 2, the majority of cases 21 (80.8%) received glucocorticoid therapy. Other drugs included colchicine, NSAIDs, hydroxychloroquine, rituximab, plasma exchange and pyridostigmine. At the time of writing no patient has severe resistant or progressive disease with all cases showing good therapy responses.

## 4. Discussion

Natural viral infection or vaccination have both been noted as potential triggering events for inflammatory diseases for many decades [40,41]. Herein, we combined data from three countries from large academic centers in regions where anti SARS-CoV-2 vaccination programs rolled out with an estimate catchment population of 6–7 million subjects. We report the nature of flares in known IMDs that generally responded well to corticosteroid therapy. We reported less common, but notable, new-onset IMDs temporally associated with vaccination administration, including neurological disease, which represents the main strength of the present investigation.

Overall, these findings of new-onset disease appear rare considering the level of population exposure in the regions covered and are likely to be close to the background incidence of these conditions in such a large population. While we cannot estimate frequency with precision, the level of public and medical vigilance around this new healthcare intervention would suggest that cases were less likely to be missed compared to, for example, seasonal influenza. Further studies would be required to compare the frequencies of IMDs after these vaccines.

Despite being rare, the insurgence of AEFIs represents a public health concern in that, if not dealt with rapidly and properly, they could undermine public confidence and trust towards vaccination. COVID-19 immunization seems to be extremely a valid strategy to contain and mitigate against the burden imposed by the pandemic and, in order to reach herd immunity, high compliance rates are warranted. Acceptance rate towards COVID-19 immunization is highly variable, ranging from approximately 90% to less than 55%, depending on the country, with information being disseminated and received playing a major role in achieving optimal coverage and community immunity [42]. Therefore, COVID-19 vaccine safety assessment is extremely crucial, especially at the beginning of population-level implementation. Our data assist clinicians in recognizing putative flares that are in line with our reported experience. The outcomes provide assurance to the public that such events are rare and manageable, and to clinicians that conventional therapies such as glucocorticoids are usually adequate—particularly important at a time where healthcare resources are highly stretched.

Numerically, our findings show the clearest associations for the non-specific adjuvant effects of the mRNA vaccines in triggering a host of different inflammatory disorders. It is possible that there is increased vigilance towards, and documentation of non-arthritis RMDs, since these are more likely to require specialist review, other investigations, or hospital treatment. Nevertheless, the proportion of the events observed is disproportionate to the usual frequency of these diseases. Usual prevalence would predict RA most commonly, followed by other inflammatory arthritis, and, then, non-arthritis RMDS. Here, we found that almost half of the events occur as forms of non-arthritis RMDs—usually far less frequent than inflammatory arthritis. Within inflammatory arthritis, spondyloarthropathy-spectrum disease was more frequent than RA, again differing from the usual prevalence, while one RA case had a usually-rare extra-articular feature. This suggests, although does not prove, causality. Most of the reported disease were flares, which supports the idea of the delicate balance of immune homeostasis in such cases being momentarily tipped into a pro-inflammatory state by vaccination.

The majority of patients in our study received mRNA vaccines while only two received DNA-based vaccine. This may simply reflect the roll-out of these vaccines rather than a causal association with IMDs. However, of particular importance, the use of TLR-7 and TLR-9 agonistic based vaccines which stimulates immunity in different ways to the more TLR-4 and inflammasome based alum vaccines represents a new vaccine strategy for human disease [43]. Most of our cases, but not all, responded very well to therapy and in most cases second doses of vaccines were withheld.

Natural SARS-CoV-2 infection has resulted so far in over 3.1 million deaths due to severe viral pneumonia where immune hyper-activation also contributes to mortality as shown by the response to some patients to corticosteroids. The most well-recognized RMD-like feature of natural SARS-CoV-2 infection is the various cutaneous vasculitis-like feature including “COVID toes” and the Multisystem Inflammatory Syndrome in Children (MIS-C) [44]. Of note, these manifestations are generally seen in subjects with minimal or not lung disease pointing towards a hyperactive immune response which, while being protective against pneumonia, can lead to collateral damage to other organ systems. Natural infection has also been associated with case reports of vasculitis, neurological disease including GBS and others in addition to other occasionally reported IMDs [45,46,47]. Herein, we report a wide variety of vaccine-associated flares or new-onset in IMDs ranging from autoinflammatory, mixed pattern disease (BD) and autoimmune diseases rather than an enrichment in autoimmune diseases that are linked to disordered nucleic acid metabolism and abnormal interferon-stimulated gene signatures involving the TLR-7/9 pathways [43].

Our findings show the presence of concomitant inflammatory arthritis and usual skin rashes in some cases. It is well established that younger subjects in particular, without severe COVID-19, experienced chilblain and erythematous lesions of the toes which had associated elevations in Type-I interferon in the skin contributing to a type of vasculitis. Of note, the TLR-7 and TLR-9 stimulation afforded by these new generations of vaccines might be expected to upregulate interferon-stimulated genes (ISGs) and contribute to robust early innate immune responses with robust type-I IFN responses thus accounting for the cutaneous and potentially other features. Dysregulated nucleic acid metabolism is associated with interferonopathies and SLE and other ANA-associated phenotypes. The frequency of such diseases is low in our series, although being similar to our rate of ACPA-positive RA this may be considered disproportionately high. We noted one case of discoid SLE converting into a systemic phenotype, with pleuro-pericarditis shortly after the TLR-9 agonist containing DNA vaccine, one case of SLE that had low activity, but where rituximab was deferred, to enhance vaccine efficacy and where patient flared following the DNA vaccine and a third case of dermatomyositis rash flare following an RNA vaccine. Monogenic interferonopathies are known to often include chilblains. Reassuringly, autoimmune myositis was not documented which is noteworthy given the intramuscular route of vaccine administration. Other relevant negatives were an absence of Macrophage Activation Syndrome (MAS)-like patterns of disease such as Adult-Onset Still’s Disease (AOSD) and undefined hyperinflammatory states mimicking MIS-C, although it is acknowledged that the latter is a disease of children and young adults, none of whom were vaccinated in the present study.

In the existing literature, only few anecdotal side-effects have been reported, including oral, oro-facial, and allergic reactions [48,49,50]. Of note, cases of Bell’s palsy, and swelling of the lips, face or tongue associated with anaphylaxis as well as flares of RA were reported [50,51]

In addition to the non-specific vaccine adjuvant properties triggered reactions as discussed above [52,53,54,55], anaphylactic reactions to BNT162b2 and mRNA-1273 based vaccines have been reported, allegedly attributed to adjuvants, like the polyethylene glycol (PEG) 2000 present in the lipid film of the two vaccine products or the polysorbate 80 utilized as excipient formulation in the preparation of the ChAdOx1 nCoV-19 vaccine [48,49,50].

In the present study, we also noted flares in BD. However, elevations in IFN levels and especially the use of recombinant IFN-alpha may have a therapeutic role in BD, although the mechanism of action of IFN-alpha in BD is not fully understood and there may be pathogenic heterogeneity.

Despite its strength, our study has several limitations, that should be properly acknowledged. From the design of this non-systematic accrual and reporting of IMDs in this early phase of the COVID-19 vaccine roll-out it is impossible to compute AEFI incidence rate or be certain of causality, even though the number of cases, their unusual presentations and apparent de-challenge response from the time of vaccine administration raise the possibility of their relationship. Other possibilities include that some of the cases actually could have had concomitant SARS-CoV-2 infection but the clinical picture without respiratory symptoms does not support this. It is also possible that some cases had prior COVID-19 disease which could have contributed to immune responses and we did not test for prior SARS-CoV-2 immunity as assessed by antibody responses. Summarizing, multiple confounding factors including concomitant SARS-CoV-2 infection and multiple co-morbidities in some cases may be contributory factors to the observed symptomatology. Given the nature of the data collection it is impossible to know how common IMD new-onset or flare of existing IMDs are and it is possible that these findings are coincidental. Nevertheless, some unusual features including concomitant rashes with IMDs that have been reported with SARS-CoV-2 infection suggest a link between vaccination, at least, in some subjects.

In conclusion this is the first large series description of IMDs flares or new-onset temporally associated with SARS-CoV-2 vaccination. The majority of cases had disease that quickly settled with corticosteroid therapy. Larger high-quality, prospective systematic studies are needed to address the issue of COVID-19 vaccination. Finally, we did not come across any cases of suspected immunothrombosis although we interacted with haematologists but it is noteworthy that 25/27 cases received mRNA vaccines. 

## Figures and Tables

**Table 1:** The study population basic characteristics. Abbreviations: NSAIDs: nonsteroidal anti-inflammatory drugs.

Parameter	Value
Sex Female Male	15 (55.6%) 12 (44.4%)
Age (mean ± standard deviation; median)	54.44 ± 19.20; 55
Pre-vaccine autoimmune/rheumatic history None Rheumatoid Arthritis Behçet’s disease Systemic lupus erythematosus (SLE)/SLE-like Pericarditis Others	6 (22.2%) 6 (22.2%) 4 (14.8%) 3 (11.1%) 2 (7.4%) 6 (22.2%)
Vaccine BNT-162b2 mRNA-1273 ChAdOx1	23 (85.2%) 2 (7.4%) 2 (7.4%)
2nd Vaccine Administered Not administered yet	12 (44.4%) 15 (55.6%)
Days from vaccine to flare/new-onset After 1st dose After 2nd dose	21/27 (77.8%); median 4 days [1–25 days] 8/12 (66.7%); median 4 days [1–7 days]
Flare New-onset	17 (63.0%) 10 (37.0%)
Severity Mild Moderate Severe	6 (22.2%) 14 (51.9%) 7 (25.9%)
Therapy Glucocorticoids Colchicine NSAIDs Plasma exchange Hydroxychloroquine Rituximab Local steroids Pyridostigmine Combination of drugs No additional treatment	21 (77.8%) 4 (14.8%) 3 (11.1%) 2 (7.4%) 1 (3.7%) 1 (3.7%) 1 (3.7%) 1 (3.7%) 7 (25.9%) 1 (3.7%)
Response Rapid response Spontaneous resolution Slow response Intubation	21 (77.8%) 2 (7.4%) 2 (7.4%) 1 (3.7%)

**Table 2:** Description of patients with immune disorder flare/new-onset following SARS-CoV-2 vaccine. Abbreviations: ACEi: Angiotensin-converting enzyme inhibitor; ANA: Antinuclear antibody; BID: Bis in die/two times daily; CBC: Complete blood count; CCP: cyclic citrullinated peptide; CRP: C-reactive protein; DM: Dermatomyositis; dsDNA: Double-stranded DNA; ESR: Erythrocyte sedimentation rate; IgE: Immunoglobulin E; MRI: Magnetic resonance imaging; MTX: Methotrexate; NSAID: Nonsteroidal anti-inflammatory drug; PLQ: Plaquenil; RF: Rheumatoid factor; RA: Rheumatoid arthritis; RS3PE: Remitting seronegative symmetrical synovitis with pitting oedema; SLE: Systemic lupus erythematosus; UC: Ulcerative colitis.

ID	Sex	Age	Pre-Vaccine History of Immune Mediated Disease + Medication	Date 1st Vaccine Dose	Date 2nd Vaccine Dose	Days from Vaccine to Flare/New-Onset (Number)	Immune Mediated Disease Complication	Flare (F)/New-Onset (N)	Severity	Relevant Lab Tests	Therapy	Comments on Therapy and Response	Country	Can the reaction be Explained Based on Drug Received by the Patient? (Yes/No)	Can the Reaction be Explained Based on the Underlying Co-Morbidity of the Patient? (Yes/No)	Do you Feel the Timing of the Side-Effect Compatible with the Time from Vaccine to Flare? (Yes/No)
1	F	83	Polymyalgia rheumatica since 2005 (clinical diagnosis) Hypothyroidism	January 2021 BNT162b2 vaccine	Not given yet	7 days (after dose 1)	Severe Seronegative Polyarthritis with RS3PE pattern (clinical assessment)	N	Severe (articular swelling and substantial limitation of range of motion)	CRP 74mg/L; ANA screening (including anti-dsDNA) negative; Anti-CCP negative; RF negative	Prednisolone 15mgs day	Rapid response to therapy with quick symptom improvement	UK	Yes	No	Yes
2	F	42	Transient synovitis (one episode in 2015, clinical diagnosis)	January 2021 BNT162b2 vaccine	Not given	4 days (after dose 1)	Migratory arthritis small joints with erythema and hemorrhagic rash on toes (clinical assessment)	F	Moderate (articular swelling and moderate limitation of range of motion)	CRP < 4 mg/L ANA screening (including anti-dsDNA) negative; RF negative	Prednisolone 10 mgs day	Rapid response to therapy with low grade swelling of 4 joints without tenderness after 1 week	UK	Yes	No	Yes
3	M	43	Neurosarcoidos is and small fiber neuropathy, diagnosed 3 years prior to vaccination. treated with infliximab for the last 2.5 years and asymptomatic under treatment	February 2021 BNT162b2 vaccine	February 2021	3 (after first dose)	Flare in neurosarcoidosis and neuropathy	F	Moderate	MRI of the spine did not show relapse of myelitis	No additional treatment	Flare spontaneously improved after 2 weeks, the patient received 2nd dose of vaccine with no additional side effects	Israel	No	Yes	Yes
4	M	38	Hypertension; dyslipidemia, and hypertrophic cardiomyopathy, treated with beta-blockers, ACEi, and atorvastatin	January 2021 BNT162b2 vaccine	January 2021 BNT162b2 vaccine	Onset (4 days after 1st dose) and flare (4 days after 2nd dose)	Pericarditis	N	Moderate	CRP 45, ESR 60. Negative for ANA	Treated with NSAIDs and colchicine. (1st event) and 20 mg prednisone (2nd event)	Rapid response to therapy with quick symptom improvement	Israel	No	No	Yes
5	M	28	Ulcerative Colitis (since 10 years) + hypereosinophilic syndrome (since 5 years) (Vedoliziumab 300 mg every 8 week and cyclosporin 150 mg BID) well controlled for a year	January 2021 BNT162b2 vaccine	Not given	4 days after 1st dose	Flare of hypereosinophilic syndrome and UC with vesicular skin rash, oral aphthosis and hemorrhagic diarrhea	F	Severe	IgE 41400, 9000 eosinophils, CRP 35	1 gr of sulomedrol daily for 3 days, then prednisone 60 mg/day tapering dose (decrease 20 mg every 2 weeks)	Slow response, eosinophils of 1200 after 2 weeks of therapy, significant and partial improvement in diarrhea and skin rash, respectively.	Israel	No	Yes	Yes
6	M	70	No medical history	December 2020 BNT162b2 vaccine	Not given	3 days after 1st dose	Polymyalgia rheumatica; severe stiffness and pain in shoulders and hips, fever and fatigue	N	Severe	CRP 175, ESR 90 Anti-CCP, RF and ANA negative	Prednisone dose 40 mg once daily	Rapid response to therapy with quick symptom improvement	Israel	No	No	Yes
7	F	45	Hypothyroidism controlled with Eltroxin 50 mcg daily	December 2020 BNT162b2 vaccine	Not given	7 after the 1st dose	Multiple sclerosis with left leg weakness disequilibrium, and lower limbs distal numbness	N	Moderate	Normal CBC and biochemistry Multiple Peri-ventricular white matter changes on MRI. CSF-oligoclonal bands.	1 g of sulomedrol daily for 5 days, then prednisone 60 mg daily with tapering down dose	Rapid response to therapy with quick symptom improvement	Israel	No	No	No
8	F	66	Idiopathic pericarditis, anemia normocytic normochromic, left femoro-popliteal DVT treated with rivaroxaban 20 mg 12.2020, spontaneous abortion of 1st trimester Idiopathic pericarditis	January 2021 BNT162b2 vaccine	Not given	2 days after the 1st dose	Pericarditis with fever, typical pleuritic chest pain, elevated inflammation markers No RA articular manifestation.	F	Mild (moderate chest pain, no symptoms of heart failure, no limitation of function)	Positive ACPA 5.4, RF 44, CRP 40, ESR 70	Continued Prednisone 15 mg prednisone. On 19 January 2021, dose was increased to 30 mg and 1 mg of colchicine. by physician. 15 mg of Methotrexate was started together with 5 mg of folic acid	Rapid response to therapy with quick symptom improvement.	Israel	No	No	Yes
9	F	36	Psoriasis since childhood, mild	December 2020 MRNA-1273	January 2021	10 days after 1st dose	Dactylitis of RT 3rd finger Onset of R 2nd and 3rd finger joint pain, stiffness and tightness. Also developed painful erythematous macules over palmar surface of several fingers on both hands Pain chilblains like lesions on fingers (painful)	N	Mild	Normal CBC, chemistry, ESR and CRP. Musculoskeletal US without synovitis or tenosynovitis (done 1/27, symptoms had nearly resolved)	Ibuprofen 800 mg	Rapid response to therapy with quick symptom improvement.	USA	No	Yes	Yes
10	F	48	Not relevant	January 2021 MRNA-1273	February 2021	10 days after 1st dose	Chilblains like lesions on fingers (painful) Urticarial-type lesions (resembled urticarial multiforme). 10 days after first vaccine developed itchy urticarial lesions over volar aspect of both wrists and feet, lesions evolved over thighs (symptomless) and extensor surfaces of both elbows, painful macular/nodular lesions over palmar surface both hands. Resolved in 7 days	N	Moderate	No work up	Hydrocortisone 0.5% Naproxen 500 mg	Rapid response to therapy with quick symptom improvement.	USA	No	No	Yes
11	M	21	Behcet’s disease (known for 3 years) treated with colchicine 1.5 mg daily	January 2021 BNT162b2 vaccine	Not given	5 days after 1st dose	Oral aphthous ulcers	F	Moderate	No work up	20 mg of prednisone for 1 week	Rapid response to therapy with quick symptom improvement	Israel	No	No	Yes
12	M	55	Behcet’s disease known for 20 years and well controlled treated with apremilast 30 mg BID. Also CLL (known for 5 years, controlled with no therapy)	December 2020 BNT162b2 vaccine	January 2021 BNT162b2 vaccine	7 days after 2nd dose	Oral aphthous ulcers and synovitis of small joints (7 days after the second dose, synovitis in MCPS and PIPS both hands, and a few days later ulcers on the tongue)	F	Moderate	No work up	Colchicine 0.5 mg twice daily and 5 mg prednisone	Rapid response to therapy with quick symptom improvement	Israel	No	No	Yes
13	M	20	Behcet’s disease (known for 2 years) treated with colchicine 1 mg daily	December 2020 BNT162b2 vaccine	Not given	3 days after 1st dose	Oral aphthous ulcers	F	Moderate	No work up	Increased dosage of colchicine to 2 mg daily	Rapid response to therapy with quick symptom improvement	Israel	No	No	Yes
14	F	62	Patient with DM treated with MTX and PLQ 200 mg BID	January 2021 BNT162b2 vaccine	February 2021 BNT162b2 vaccine	7 days after 1st dose	Skin rash similar to the rash when diagnosed with DM	F	Mild	No work up	Local steroid cream	Flare spontaneously improved after a week, the patient received 2nd dose of vaccine with no additional side effects	Israel	No	No	Yes
15	F	70	RA, treated with MTX 15 mg wk and Certolizumab 200 mg every other week (on remission for the last 18 months). Medical history included hypertension	December 2020 BNT162b2 vaccine	January 2021 BNT162b2 vaccine	7 days after 2nd dose	Polyarthritis of small joints	F	Moderate	High CRP	NSAIDs	Rapid response to therapy with quick symptom improvement	Israel	No	No	Yes
16	F	78	Laboratory SLE with no clinical manifestation	January 2021 BNT162b2 vaccine	Not given	2 days after 1st dose	Fever, erythematous rash (generalized acute cutaneous lupus), purpura, oral aphthous ulcers and arthritis	N	Mild	+ANA, high CRP and ESR, Skin biopsy from purpura was compatible with leukocytoclastic vasculitis	Started on Hydroxychloroquine	Rapid response to therapy with quick symptom improvement	Israel	Yes	No	Yes
17	F	50	SLE with arthritis, mucosal ulcers and hemolysis (2019 EULAR/ACR SLE criteria)	January 2021 ChAdOx1 nCoV-19 vaccine	Not given	14	Severe hemolysis, oral and nasal ulceration, arthralgia	F	Severe (clinical assessment)	Hb 53, reticulocytes 42%, bilirubin 48, LDH 864	High dose glucocorticoids (Prednisolone 60 mg daily) and rituximab	Slow response	UK	Yes	No	Yes
18	M	34	Behcet’s disease (known for 10 years, included arthritis, uveitis, skin rash, oral aphthosis controlled for the last 12 months, treated with colchicine and Humira), no other medical history	January 2021 BNT162b2 vaccine	Not given	5 days after 1st dose	Pustular skin lesions -developed skin lesions as a part of Behcet’s disease for the first time	F	Moderate	No work up	Short course of NSAIDs and increase of colchicine dose	Rapid response to therapy with quick symptom improvement	Israel	No	No	Yes
19	M	72	Recurrent. pericarditis - treated with colchicine in the past	January 2021 BNT162b2 vaccine	January 2021 BNT162b2 vaccine	1 day after 2nd dose	Myasthenia Gravis	N	Moderate	EMG-decrement of 28–46% on facial and shoulder muscles	Plasma exchange + Prednisone 60 mg	Rapid response to therapy with quick symptom improvement	Israel	No	No	Yes
20	M	73	Not relevant	Dec 2020 BNT162b2 vaccine	January 2021 BNT162b2 vaccine	7 days after 2nd dose	Myasthenia Gravis (firstly ocular signs then appearance of bulbar signs and respiratory symptoms)	N	Severe	EMG- borderline decrement, markedly pathologic jitter	Plasma Exchange, Pyridostigmin and prednisone	Patient intubated due to respiratory symptoms	Israel	No	No	Yes
21	F	68	RA seropositive-well controlled with Actemra	January 2021 BNT162b2 vaccine	February 2021 BNT162b2 vaccine	3 days after 2nd dose	Flare of synovitis of small joints CDAI = 41 compared to CDAI = 4 in 19 January 2021	F	Severe	ESR=12 after depomedrol, CRP = 0.06 before flare, CRP = 0.28 after flare, RF = 1782 before flare, RF = 2300 after flare, ANA negative after vaccine	IM Depomedrol 160 mg	Rapid response to therapy with quick symptom improvement	Israel	No	No	Yes
22	M	53	Not relevant	January 2021 BNT162b2 vaccine	Not given	3 days after first dose	Palpable purpura, mild abdominal pain and arthralgia w/o arthritis, no renal involvement. Diagnosed with Henoch Schlonein Pupura with Leukocytoclastic cutaneous vasculitis among skin bipsy	N	Mild-moderate	Skin biopsy revealed IgA and C3 deposits in the vessel walls Mild elevation in CRP to 0.8 mg/dL, mild elevation in IgA to 412 mg%, mild leucocytosis to 11.5 k/ul	Dexamethasone 10 mg, prednisone 60 mg thereafter	Rapid response to therapy with quick symptom improvement	Israel	No	No	Yes
23	F	80	Seronegative RA–2007 (ACR/EULAR 2010 class. Crit.) stable on Methotrexate 15mgs week	January 2021 BNT162b2 vaccine	Not given	25 days flare with progressive worsening	Severe Right ankle synovitis, severe wrist synovitis and 5 small joint synovitis	F	Severe (articular swelling and substantial limitation of range of motion)	ESR 71 CRP 27	Prednisolone 15 mgs day tapering and increase methotrexate dose	Rapid response to therapy with quick symptom improvement	UK	No	No	Yes
24	M	70	Gout (known for many years) well controlled in the last 2 years), hypertension and dyslipidaemia	Dec 2020 BNT162b2 vaccine	January 2021 BNT162b2 vaccine	1 day after first dose and another flare 1 day after the second dose	Monoarthritis at Rt elbow (after 1st dose) and at mid foot (after 2nd dose)	F	Moderate	CRP 44 mg/L.	Prednisone 40 mg for 7 day	Rapid response to therapy with quick symptom improvement	Israel	No	No	Yes
25	F	78	Seropositive RA (not active and with no therapy for many years)	January 2021 BNT162b2 vaccine	Not given	2 days after first dose	Polyarthritis involving shoulders, wrists, MCPs and PIPs	F	Moderate	ANA negative, RF 60, ESR 52, CRP 2.1 mg/dl	Prednisone 20 mg daily with tapering down 5 mg every week was prescribed.	Rapid response to therapy with quick symptom improvement	Israel	No	No	Yes
26	F	27	Seronegative RA stable on Adalimumab every 2 weeks	January 2021 BNT162b2 vaccine	January 2021 BNT162b2 vaccine	4 days after second dose developed Rt knee monoarthritis	Monoarthritis of Rt knee	F	Moderate	ANA negative, RF negative, CRP 1.3 mg/dl	Joint aspiration and injection of 80mg depomedrol	Rapid response to therapy with quick symptom improvement	Israel	No	No	Yes
27	F	60	SLE (never previously referred to rheumatology): Discoid lupus since 2001, severe raynauds, alopecia, pancytopenia (since September 2020), proteinuria (Developed Sept 2020, diagnosed as class IV lupus nephritis March 2021), hypocomplementaemia, ANA positive, dsDNA, anti-chromatin and anti-ribosomal antibody positive), 2019 EULAR/ACR SLE criteria) Baseline Disease modifying therapy: Prednisolone 8mg OD	February 2021 ChAdOx1 vaccine	Not given yet	4 days	Pericardial and pleural effusions, possible pericarditis	F	Mild	Central intermittent pleuritic chest pain, had CTPA and bedside echo. Trop normal CRP 17.8	Prednisone increased from 8mg to 15mg for 5 days, colchicine 500 mcg BD	Rapid response to therapy with quick symptom improvement	UK	Yes	Yes	Yes

## Data Availability

All data generated are contained in the present manuscript.
